# Prevalence of obesity in treated and untreated patients with attention deficit hyperactivity disorder: A meta-analysis

**DOI:** 10.15537/smj.2022.43.8.20220247

**Published:** 2022-08

**Authors:** Fatimah AlAhmari, Mohy Uddin

**Affiliations:** *From the Pediatrics Department (AlAhmari), King Faisal Specialist Hospital and Research Centre; and from the Research Quality Management Section (Uddin), King Abdullah International Medical Research Center, King Saud bin Abdulaziz University for Health Sciences, Ministry of National Guard - Health Affairs, Riyadh, Kingdom of Saudi Arabia*

**Keywords:** attention deficit, hyperactivity disorder, ADHD, psychostimulants, obesity, overweight

## Abstract

**Objectives:**

To discuss and summarize the scholarly published literature on the difference in obesity rate in treated and untreated attention deficit hyperactivity disorder (ADHD) patients to evaluate the influence of ADHD medication on weight status in these individuals.

**Methods:**

PubMed, Cochrane Library, and Google Scholar databases were searched for eligible articles from January to December 2020 using the following medical subject headings (MeSH) terms: “attention deficit hyperactivity disorder and other hyperactivity disorders”, “obesity and overweight”, “obesity treatment”.

**Results:**

A total of 19,449 study participants included in selected 8 studies were assessed with respect to the prevalence of obesity in medicated and unmedicated subgroups of ADHD patients. The total number of ADHD patients with the prescribed medication was 10,279, while the number of unmedicated ADHD patients was 9,170. The odds ratio was 0.65 with a 95% confidence interval of 0.50 to 0.84 favoring regular medical treatment for management of obesity in case of patients with ADHD.

**Conclusion:**

The prevalence of obesity observed in treated ADHD patients was significantly lower compared to that in unmedicated patients. This result suggests that the treatment is not only important for controlling ADHD manifestations but is also associated with lower body mass index. Therefore, further prospective studies with large sample size are required for controlling the confounding factors such as comorbidities and medication status.

**P**hysical health problems are frequently observed in mental health patients compared to the general public.^1^ Previous studies have confirmed that somatic symptoms significantly contribute to mental health symptoms, and comorbidities play an important role in aggravating primary symptoms, causing physiological impairment, lowering life quality, and enhancing economic burden.^2-5^ While studying comorbidities of psychiatric and medical diseases, Druss et al^6^ concluded that approximately 68% of psychiatric patients had one or more somatic disorders. The interlinking of mental and somatic disorders has been understood to a certain extent. For example, mood disorders are seen in hypothyroidism, and schizophrenia and bipolar disorders are associated with metabolic syndrome probably due to that the effect of medication on the development of these complications overrides lifestyle factors.^7-9^ Moreover, it has been well-observed that most psychiatric patients tend to have unhealthy and unhygienic dietary habits and a sedentary lifestyle.^10^

Attention deficit hyperactivity disorder (ADHD) has been found to be the most frequent childhood-onset neurodevelopmental disorder, according to the American Psychiatric Association’s DSM-5, it as the presence of 6 or more symptoms in either the inattentive or hyperactive and impulsive domains, or both, that imposes an enormous burden on the population.^11,12^ It has been reported that impairing symptoms of ADHD in children can persist in adulthood in up to 65% of individuals with a prevalence of ADHD in adulthood of almost 2.5%.^13^ The study from Jensen et al^14^ found that 87% of ADHD cases had one comorbidity, and 67% of ADHD cases had more than one comorbidity.Similarly, Bidermanet et al^15^ stated that 20% of ADHD cases attending clinics had at least 2 comorbidities.All comorbidities seen in ADHD patients could be a manifestation of a single disorder in different ways, or conversely different disorders may have a similar appearance. Therefore, ADHD may be an early manifestation of some other disease or may indicate the development of another disease.^15^ Specifically, there has been an increasing amount of evidence on possible association between ADHD and obesity/overweight; this association is important when considering ADHD treatment.^16,17^ The available treatments for ADHD include pharmacological treatments with mainly psychostimulant medications such as methylphenidate, amphetamines, and bupropion.^13,18^ Most common side effects of these treatments are decreased appetite, headache, abdominal pain, sleep disturbances, and irritability, and less frequent side effects include weight loss, social withdrawal, tics, and emotional modifications in some cases.^19^

A longitudinal clinical intervention was carried out in 2009 to assess the effects of ADHD drugs on weight change by following 78 severely obese adults diagnosed with ADHD (65 received medication for ADHD and 13 were controls) for 466 days. Its findings revealed that ADHD medication is associated with significant long-term weight loss in patients with a previous history of weight loss failure (12.4% weight loss of initial weight in treated patient versus 2.8% weight gain in untreated patients).^20^ Similarly, another recent meta-analysis conducted by Cortese et al^21^ reported that patients receiving medications for ADHD were not at a higher risk for obesity, which was not the case in untreated ADHD patients. Therefore, these studies suggest the importance of stimulant treatment in both managing ADHD symptoms and improving nutritional status in obese ADHD patients.

To elucidate the effect of stimulant pharmacological treatments on nutritional status and weight, it is important to examine the impact of these drugs on ADHD obese patients. The aim of this meta-analysis is to discuss and summarize published scholarly literature on the difference in obesity rate in treated and untreated ADHD patients to evaluate the effect of ADHD medication on weight status in these individuals. The objective is to answer the following question: “Is there a difference in the prevalence between medicated and unmedicated ADHD patients?”

## Methods

This study was performed in accordance with the Preferred Reporting Items for Systematic Reviews and Meta-Analysis (PRISMA) guidelines. All studies evaluating the prevalence of obesity in individuals with ADHD were considered to be eligible for inclusion in the meta-analysis. The studies that mentioned the body mass index (BMI) or obesity status of children aged under 18 years, suffering from obesity in association with ADHD with or without prescribed concomitant stimulant medications were also included in the meta-analysis. The following points were considered as the inclusion criteria: studies that evaluated prevalence of obesity in the ADHD population, studies that included medicated and unmedicated ADHD patients, studies that mentioned the BMI status of study participants or obesity in general, and studies with a parallel study design that enrolled both medicated and unmedicated patients suffering from ADHD who were overweight and at risk of developing obesity or who were diagnosed with obesity. The exclusion criteria consisted of: studies that included patients above 18 years of age, studies that included medicated or unmedicated ADHD patients without the other corresponding arm, studies that considered the health outcomes of patients with ADHD but were lacking any comments with respect to body weight and BMI, and studies mentioning the prevalence and control of obesity in individual suffering from autism spectrum disorders (ASD) and other psychological and psychiatric conditions apart from ADHD. The majority of studies that mentioned the prevalence of obesity were cross-sectional and later performed subgroup analysis to ascertain the prevalence and control of obesity in medicated and unmedicated individuals suffering from ADHD along with a few case reports limited to individual findings. Studies that satisfied the selection criteria but were available in the form of abstracts, incomplete studies (such as, those with missing results), duplicates, and unpublished articles were not considered for inclusion in the meta-analysis. Finally, all eligible articles available in English language were selected for evaluation.

Search terminology was defined according to the protocol for identification of the search terms with respect to the prevalence of obesity in medicated and unmedicated individuals with ADHD. The search terms were used with the Boolean operator “AND” to filter the related articles. Free text search utilized the words such as ADHD, treatment, medicated, and unmedicated along with the Boolean operator “OR” in-between them and in association with BMI, obesity, and overweight with the Boolean operator “AND”. In contrast, the controlled language search included the following medical subject headings terms: “attention deficit hyperactivity disorder and other hyperactivity disorders”, “obesity and overweight”, and “obesity treatment”. PubMed, Cochrane Library, and Google Scholar databases were searched for eligible articles from January to December 2020. The reference list of all included studies was further reviewed to include the eligible studies. During the database search for potential studies, titles and abstracts of the articles were screened according to the selection criteria by independent reviewers.

Data was extracted individually and recorded in a customized data extraction sheet by 2 independent reviewers. Discrepancies regarding the inclusion of studies were resolved through discussion or arbitration by involving an additional reviewer, as required. Once the selection of preliminary studies was carried out based on the criteria, full texts of the articles were assessed to evaluate the study content for the meta-analysis. Information pertaining to the prevalence of obesity and the effect of medications on the BMI control was extracted and analyzed. The risk of publication bias assessment was independently performed on each study by 2 reviewers. The overall search process resulted in 1,131 articles, which were reduced to 319 after the removal of duplicates. Eligibility screening yielded 29 studies that were then subjected to full text review. Finally, 8 articles were selected for the meta-analysis.

### Statistical analysis

A descriptive analysis of the extracted data was performed. Similar findings from the studies were identified and quantitatively analyzed, which were then represented accordingly. The meta-analysis was performed using the Revman software version 5.3.^22^ To address the heterogeneity of the included studies with respect to the study population, study environment, and trial setting (which may differ in different clinical trials), the findings from individual studies were pooled using the random effects model, which helped to identify potential areas that could significantly alter the results. Forest plot showing the odds ratio (OR) of the point estimates along with 95% confidence interval (CI) for the prevalence of obesity in medicated and unmedicated individuals with ADHD was constructed and represented accordingly. The degree of heterogeneity among the included studies was evaluated using the I^2^ test, which acts as a tool to understand the degree to which the studies differ in their clinical trial setting.

## Results

**Figure 1:**
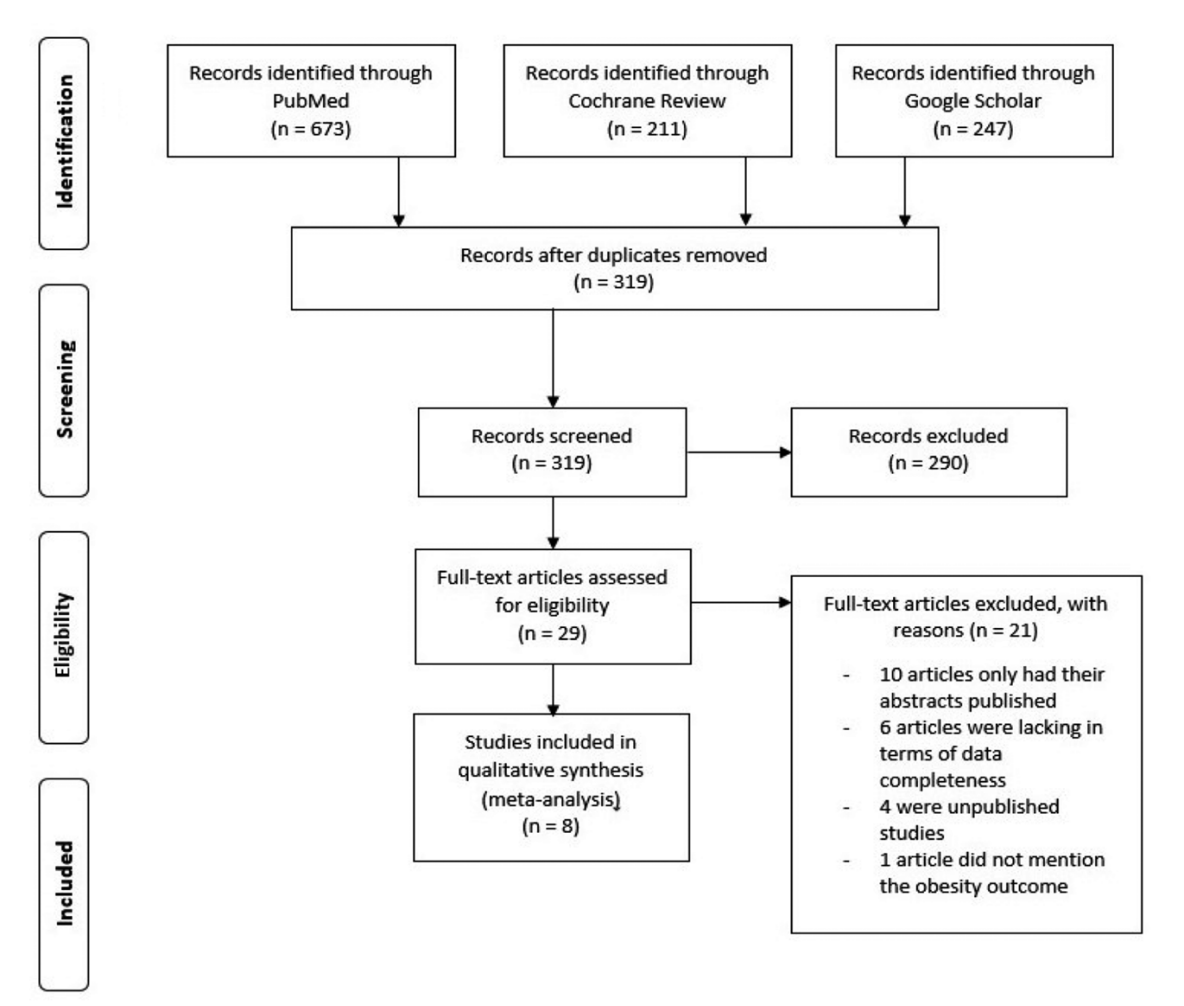
- Preferred Reporting Items for Systematic Reviews and Meta-Analysis flowchart diagram.

After the search and screening process by 2 independent reviewers, a total of 8 studies were selected for meta-analysis.^1-8^ The details of identification, screening, eligibility, and inclusion of studies is shown in PRISMA flowchart in Figure 1.

A total of 19,449 study participants included in those 8 studies were assessed with respect to the prevalence of obesity in medicated and unmedicated subgroups of ADHD patients. The total number of ADHD patients with prescribed medication was 10,279, while the sum of unmedicated ADHD patients was 9,170. A total of 359 obese patients were in the medicated subgroup, while 509 obese patients were in the unmedicated group. Five studies^1-5^ discussed the prevalence of obesity by considering BMI as the criteria for distinction between normal weight and overweight, while remaining 3 studies only described the people who were at risk for developing obesity. The characteristics of the included studies are listed in Table 1.

**Table 1:** - Characteristics of the included studies.

Author (year)	Title	Design	No. of study participants (medicated / unmedicated)	Gender distribution(boys/girls) and race	Age group	BMI range and obesity criteria used	Type of medication used for ADHD
Curtin et al^24^ (2005)	Prevalence of overweight in children and adolescents with attention deficit hyperactivity disorder and autism spectrum disorders: a chart review	Retrospective chart review	98 (32 / 66)	(79 / 19)Whites, African American, Hispanics	3 to 18 years	Obesity was defined as >95% percentile of the CDC growth charts	Stimulant medications (methylphenidate and amphetamine salts) used
Visser et al^25^ (2007)	National estimates and factors associated with medication treatment for childhood attention-deficit/hyperactivity disorder	National Survey of Children’s Health data	6497 (3746 / 2751)	(4683 / 1814)Hispanic and Latino	4 to 17 years		Stimulant medications used
Waring and Lapne^26^ (2008)	Overweight in children and adolescents in relation to attention-deficit/hyperactivity disorder: results from a national sample	Cross-sectional study	5680 (2424 / 3256)	(4101 / 1579)Hispanic and non-Hispanic blacks and whites	5 to 17 years	Obesity was defined as >95% percentile of the CDC growth charts	NA
Dubnov-Raz & Berger^27^ (2011)	BMI of children with attention-deficit/hyperactivity disorder	Retrospective chart review	275 (135 / 140)	NA Jewish	6 to 16 years old	Overweight and obesity was defined as >85% and >95% percentile of the CDC growth charts	Stimulant medication (methylphenidate) used
Kim et al^28^ (2011)	Health behaviors and obesity among US children with attention deficit hyperactivity disorder by gender and medication use	Cross-sectional study	6070 (3580 / 2490)	(4356 / 1714)Hispanic and African American	6 to 17 years	Obesity was defined as >95% percentile of the CDC growth charts	Stimulant medications used
Byrd & Curtain^29^ (2013)	Attention-deficit/hyperactivity disorder and obesity in US males and females, age 8-15 years: National Health and Nutrition Examination Survey 2001-2004	National Health and Nutrition Examination Survey	412 (185 / 227)	(275 / 137)Non-Hispanic whites and blacks Mexican Americans	8 to 15 years	Obesity was defined as >95% percentile of the CDC growth charts	NA
Spencer et al^30^ (2014)	Attention-deficit/hyperactivity disorder and adverse health outcomes in adults	Cross-sectional study	198 (98 / 100)	(93 / 105) Caucasian	8 to 18 years	NA	Stimulant medications used
Hanć et al^31^ (2015)	ADHD and overweight in boys: cross-sectional study with birth weight as a controlled factor	Cross-sectional study	219 (79 / 140)	All 219 boys Polish	6 to 18 years	NA	Stimulant medications used

ADHD: attention deficit hyperactivity disorder, US: United States, NA: not available, &: and, BMI: body mass index, CDC: Centers for Disease Control and Prevention, No.: number

### Quality assessment of included studies

The quality of included studies was evaluated using the STROBE guidelines for observational studies.^23^ Special emphasis was given to the study aim, study participants, clinical trial design, parameters used for assessment of the primary objective, inclusion and exclusion criteria applied for selection of the study sample, treatment arms, variables used for defining the outcome, exposure, and potential confounders and predictors. Methodological quality of the included studies was evaluated to minimize the probability of bias at different points during study selection. The majority of studies scored well in all above mentioned domains; however, there was no formal sample size calculation mentioned in any of the included studies, which limited the overall scoring of all included studies to below 75%.

### Prevalence of obesity in ADHD patients

**Figure 2:**
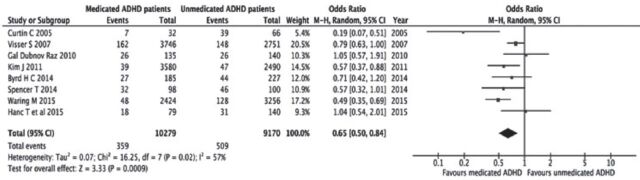
- Forest plot-prevalence of obesity in medicated versus unmedicated attention deficit hyperactivity disorder (ADHD) patients. CI: confidence incterval

The overall summary estimate of the forest plot (Figure 2) favored the medicated ADHD group to have a low prevalence of obesity.

Unmedicated ADHD patients were more prone to develop obesity compared to those on treatment for ADHD. The prevalence of obesity was 4 per 100 patients in the medicated subgroup of ADHD (3.5%), while in the case of patients who did not receive any treatment for ADHD, 6 out of 100 patients suffered from obesity (5.6%). The majority of studies recommended adequate medications to be the key factor for obesity control, while 3 studies indicates that ADHD patients with prescribed medications were at equal risk for developing obesity compared to their unmedicated counterparts.^1-8^ The *p*-value for overall summary estimate was 0.0009 along with a Z score of 3.33. The odds ratio (OR), calculated using the Mantel–Haenszel random effects model for prevalence of obesity was 0.65 with a 95% CI of 0.50 to 0.84. The combined estimate favored regular medical treatment for management of obesity in case of patients with ADHD because the 95% CI for odds ratio of the summary estimate was completely present on one side of the line of zero effect favoring medicated ADHD group.

### Exploration of heterogeneity and publication bias

**Figure 3:**
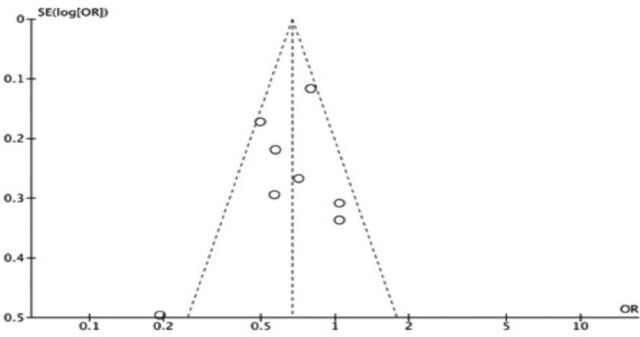
- Funnel plot-publication bias.

In addition to the I^2^ test, Egger’s test was used to assess the heterogeneity of the study. Special precautions were observed to prevent the occurrence of publication bias. The inverted funnel plot (Figure 3) showed a nearly equal representation of studies mentioning obesity to be associated with medicated and unmedicated ADHD patients.

The I^2^ test’s value of 57% indicated moderate variability among the selected studies. In addition, a major part of 95% CI of individual studies was overlapping with each other, thus representing moderate study variability.

## Discussion

Our study findings reported a prevalence rate of obesity of 3.5% in the medicated subgroup of ADHD and 5.6% in patients not receiving any treatment for ADHD. In recent years, several investigations suggested the increased prevalence of ADHD in obese patients and vice versa, and some studies have attempted to explain the reasons for this association. For example, obesity or other risk factors, including fragmented sleep and hypoxemia (due to apneas and hypopneas), may cause excessive daytime sleepiness that in turn contributes to similar ADHD signs and symptoms.^32,33^ Furthermore, Weinberg and Brumback^34^ suggested the hypoarousal theory consisting of oversleepiness of ADHD patients compared to controls. Attention deficit hyperactivity disorder patients manifested hyperactivity and impulsivity to stay awake and alert.^34^ Another study carried out to evaluate sleep and alertness in children with ADHD showed that these patients were sleepier during the day with a deficit in alertness compared to controls.^35^

Our meta-analysis revealed that the prevalence of obesity observed in treated ADHD patients was significantly lower compared to that in unmedicated ADHD patients, with OR equal to 0.65 with 95% CI of 0.50–0.84, favoring regular medical treatment for management of obesity in case of patients with ADHD. This result was possibly obtained due to the anorectic effects of methylphenidate causing reduced appetite and resulting in weight loss.^36^ A previous meta-analysis carried out by Cortese et al,^21^ which included 12 articles, had similar findings yet non-significant results, where patients treated with ADHD medications had rates of obesity that were lower by approximately 40% compared to untreated patients. In addition, the findings of another study,^19^ which aimed to evaluate the association of treatment duration with weight loss, showed that after comparing patients treated for less than one year with those treated for more than 3 years, these groups had a significant decrease in BMI, showing that BMI tended to decrease without considering the period of treatment. However, the reason for weight loss in all situations has not yet been established, specifically after knowing that the effects of methylphenidate are generally temporary and become less evident during treatment. Strimas et al^37^ and Davis et al^38^ suggested that pharmacological treatments are capable of both reducing impulsiveness/inattention and improving inhibitory control, resulting in a decrease in caloric intake with a better and organized diet plan, which in turn may have assisted in weight reduction. However, a naturalistic study by Levy et al^20^ reported that appetite suppression was evident within 1-1.5 months after starting the treatment, but then it reduced and vanished in the majority of participants within 2 months; the authors concluded that it was not likely that the temporary anorexigenic impact of psychostimulants contributed to the observed weight loss at follow-up >1 year after the start of treatment.

Attention deficit hyperactivity disorder must be presented as the main reason of weight loss failure in the obese population. Patients diagnosed and seeking medical or surgical weight loss must be assessed for ADHD and treated accordingly prior to intervention. Similarly, ADHD patients must be evaluated for possible risk of obesity during their assessment and management. This will result in an improved management outcome and reduced occurrence of post-surgical complications due to poor compliance with diet and supplement requirements.^20^

Furthermore, a previous systematic review was carried out to assess the effect of physical activity on children with ADHD on the short and long run. Results suggested clinical benefits including alleviated cognitive, behavioural and physical symptoms of ADHD due to exercising.^39^

Though there are many other databases and indexing services for peer-reviewed literature like PsycINFO, Scopus and Web of Science, but due to our study limitations, we only searched PubMed, Cochrane Library and Google Scholar. This meta-analysis has important clinical and public health implications. Assessing the risk for obesity should be part of the assessment and management of ADHD patients, especially untreated ADHD patients. However, our results showed the following limitations. The publication bias could have occurred from including only studies that were published in English. The analysis excluded unpublished studies that might have been presented at conferences. The analyzed cross-sectional data did not provide deductive conclusions on causality in the association between ADHD medications and obesity status. The definition of obesity in the included studies in children and adolescents was based on different BMI thresholds and definitions, such as Z-scores [85th (at risk for overweight) and 95th percentiles (overweight) of BMI], or BMI adjusted for gender and weight based on WHO’s growth charts and then presented as Z scores.^24,27,31^ It must be also taken into consideration a re-analysis of the original data (Individual participant data IPD) rather than just extracting aggregated data to allow further subgroups analyses. Most study participants in this analysis were obese; hence, future studies are needed to provide more information on patients with less severe grades of weight excess. Another caveat to this review was that we did not focus on duration of illness and medication use due to the limited number of studies that examined these variables. Finally, the long-term effects of psychostimulants on weight was not fully considered in the present study; weight loss may be related to direct anorexic effects of psychostimulants, or long-term normalization of eating patterns, or other indirect mechanisms.

In conclusion, over the last 3 decades, obesity has become an epidemic posing serious public health issues. In the literature, many studies have assessed the association between ADHD and obesity, and preliminary evidence revealed that the prevalence of obesity observed in treated ADHD patients was significantly lower compared to that in unmedicated patients, suggesting that treatment is associated with lower BMI. Therefore, further prospective studies with large sample size controlling for confounding factors, such as population characteristics including comorbidities and medication status must be investigated further in future research. Moreover, behavioral modification and obesity intervention programs must also take these factors into consideration.
